# Evaluation and Analysis for Chinese Medical Alliance’s Governance Structure Modes Based on Preker-Harding Model

**DOI:** 10.5334/ijic.5417

**Published:** 2020-11-24

**Authors:** Feng Yang, Yansui Yang, Zangyi Liao

**Affiliations:** 1 Institute for Hospital Management, Tsinghua University, Beijing, CN; 2 School of Political Science and Public Administration, China University of Political Science and Law, Beijing, CN

**Keywords:** medical alliance, governance model, Preker-Harding model, key incentives, medical resource allocation

## Abstract

**Objective:**

Analyze and evaluate the typical four medical alliance’s governance modes in China, and construct a set of medical alliance’s governance mode that adapt to the current status of medical resource allocation in China.

**Theory and Methods:**

We used interview-based case studies to investigate the four most representative medical alliance modes in China, and conducted in-depth analysis and discussion of key incentives affecting medical alliances under the guidance of the Preker-Harding model framework.

**Results:**

The results show that the essence of the relationship between the government as the owner and the medical alliance is the entrustment and adjustment of power and responsibility; the government as a regulator has a normative and universal regulation of the medical alliance; the reform of the medical alliance requires the government to clarify the functional positioning of the medical alliance and determine a reasonable compensation system.

**Conclusion:**

China should establish the “Positive Triangle” model of medical alliance’s governance, this medical security model provides patients with various types of medical services in a horizontal dimension, covering a variety of difficult disease treatments in a vertical dimension.

## Introduction

Medical alliance refers to a group of medical institutions or joint organizations formed by the integration of horizontal or vertical medical resources in different regions and types of medical institutions within a certain region, the medical institutions are mutually beneficial and responsible communities [1].

According to the closeness of the contact, the medical alliance can be divided into three types: compact, semi-compact and loose [1]. A compact medical alliance is an operational management mode that forms a community of responsibilities and interests among various health service organizations within a medical alliance, and all people and property implement unified operational management; The semi-compact medical alliance refers to an operational management mode formed by the contract or agreement from the core hospital to the primary health center on the basis that the nature of the assets of various medical service institutions does not change in the medical alliance; Loose medical alliance means that all kinds of health service organizations in the medical alliance only cooperate in medical technology, personnel training, equipment and other aspects. However, various medical and health institutions within the medical alliance are not affiliated.

The medical alliance is an important carrier of hierarchical medical system. Its establishment is an effective exploration and practice mode to optimize the allocation of medical resources, improve the service level of primary medical institutions, control the cost of medical and pharmaceutical services, improve the patient experience, and comprehensively improve the health management level and chronic disease management level [2,3]. The structure, process, and outcome of medical services involve multiple subjects such as patients, doctors, hospitals, government, and society. This places high demands on the allocation of medical resources. This is a comprehensive governance process. However, as the medical service environment becomes more and more complex, the traditional medical service organization mode can no longer meet the requirements of modern medical services for efficiency and social responsibility [4].

Due to the increase in medical risks and the pressure on the medical service market, integrated delivery networks (IDN), a medical resource integration model or a systematic integration change, are necessary [5]. This change will enable the quality and efficiency of medical services to be improved to some extent through synergy between different medical units [6].

**Figure 1:**
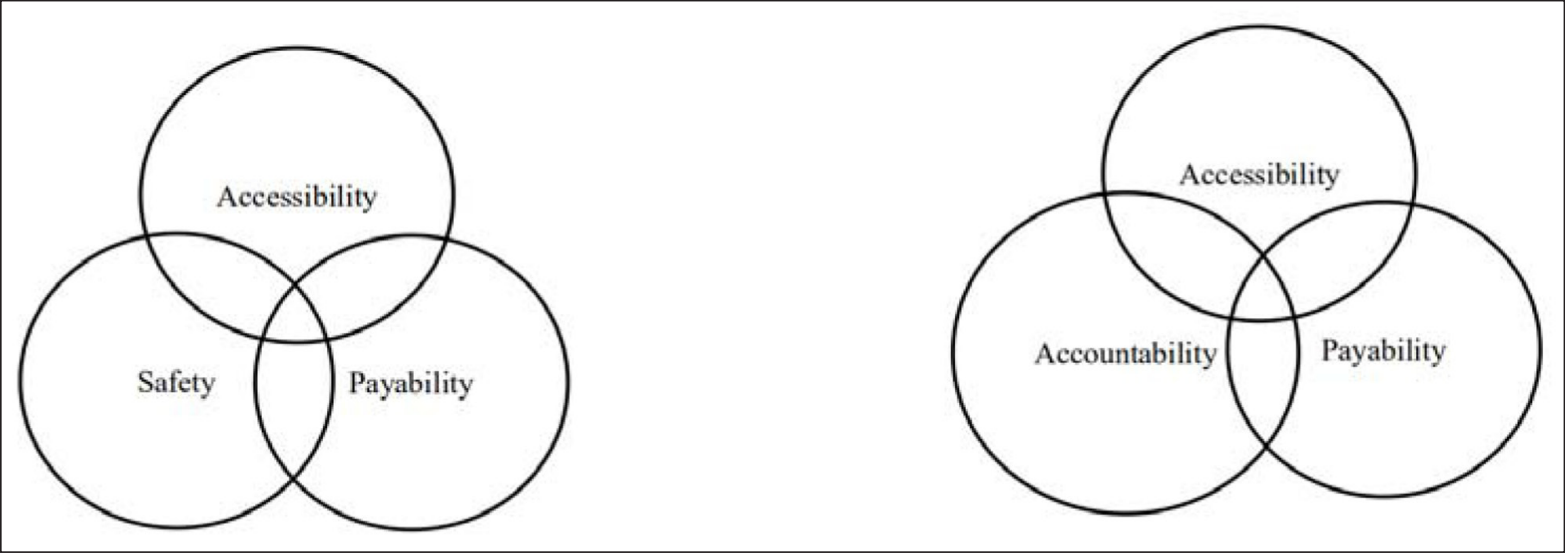
The Three Dimensions of WHO’s Health Care and PPACA’s Health Care.

In recent years, many regions and countries have explored new forms of medical alliances, such as the United States, Canada, Brazil, and Cuba. Its main purpose is to continuously improve the fairness and accessibility of health care services. For example, as early as 1992, the City of Boston launched a Community Medical Alliance (CMA) [7]. The Patient Protection and Affordable Care Act (PPACA), passed in March 2010, is mainly aimed at providing reasonable basic medical services for all people [8,9]. This is consistent with World Health Organization (WHO) universal health concept [10,11]. The core of PPACA is to pay more attention to the coverage and quality of medical services based on the three dimensions (safety, accessibility, and pay ability) of medical services proposed by WHO (Figure 1) [12].

In mainland China, As of June 2017, 80% of tertiary hospitals (1,764) have participated in the construction of medical alliances, with more than 400 medical alliances [13]. Most of these alliance types are based on the actual situation of each place, and the resource allocation method has been adjusted differently. The essential purpose and trend of these alliances are the same, that is, through the scientific and rational allocation of medical resources, the quality medical resources are promoted and connected, so as to improve the health management and chronic disease management level of residents.

It can be seen that most countries are constantly exploring and practicing the medical alliance modes. The United States’ Integrated Delivery System (IDS) is more inclined to link different levels of health care services to provide coordinated, continuous medical services to specific patient populations and community residents [14]. The UK’s Integrated Care Network (ICN) advocates the integration of resources in the health care system and provides one-stop medical services to patients through the integration of primary health care systems and social care systems, thereby ensuring the quality, cost and efficiency of services [15]. China also has multiple modes in the construction of medical alliances. However, due to the intricate and inexperienced status of China’s medical resource allocation, in the process of reform, although the medical alliance does have certain advantages in achieving referral, improving the level of primary medical services, and rationally arranging regional medical resources, because various types of medical service organizations have differences in the ownership of assets, the distribution of personnel, the orientation of interests, and the degree of independence. In addition, different medical service organizations may have contradictions in management concepts, performance appraisal principles, salary standards, hospital culture, and risk control capabilities (For example, in a loose medical alliance, because of the relative independence of staff deployment and benefit distribution, stability and sustainability are poor, and it is difficult for hospitals to form a true community of interests and responsibilities; Although the compact medical alliance realizes the unified management of property rights, the history and management style of hospitals may be different, so this requires a process of bridging business and integration), which makes the reform more difficult [16,17].

Therefore, this study aims to provide recommendations for China’s medical alliance reform by in-depth analysis and evaluation of the core mechanisms and advantages and disadvantages of China’s four major medical alliance governance modes, and also provide case references for medical resource allocation in other countries.

### Theoretical Model Perspective

**Figure 2:**
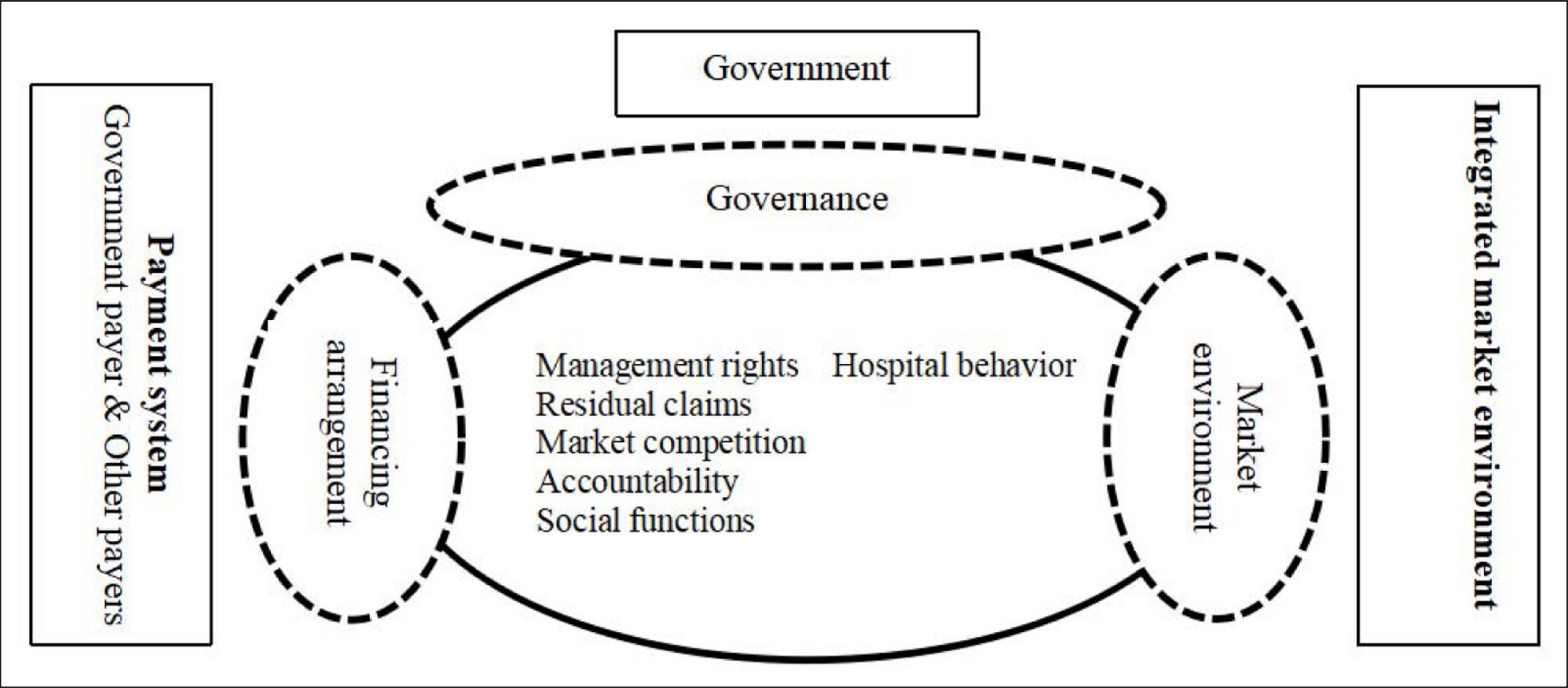
Key Determinants of Organizational Behavior Transform.

**Figure 3:**
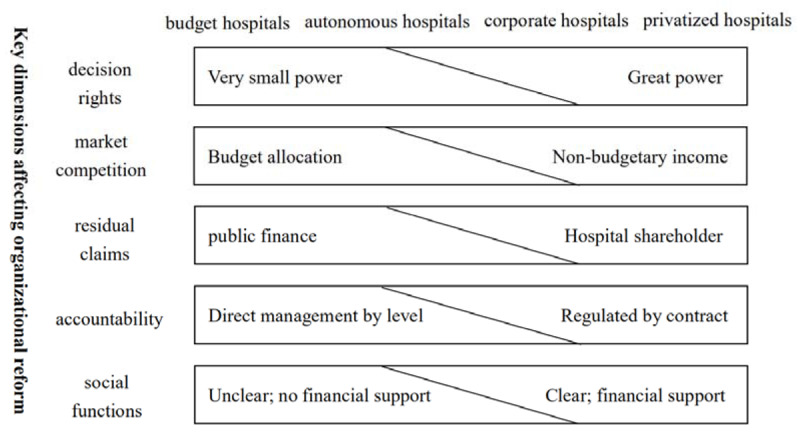
Organizational Selectable Preker-Harding Model.

This study selects the Preker-Harding model to analyze and evaluate the modes of the governance structure of Chinese medical alliance. Firstly, we analyzed the applicability of the model. The Preker-Harding model was created by World Bank economists Alexander S. Preker and April Harding in 2003 based on an analysis of public hospital reforms in many countries. The model aims to effectively analyze and evaluate the behavior and patterns of change in various public hospitals [18]. As shown in Figure 2, there are three typical system elements (governance, market environment, financing arrangements) that influence incentives and hospital behavior [19]. Governance refers to the relationship between the owner (government) and the hospital [20]; the market environment refers to the degree of multiple market pressures in which the hospital operates normally [21]; the financing arrangement refers to the structure in which funds flow from the fundraiser or the payer to the hospital [22]. According to research by Preker and Harding, these three factors depend on the combined impact of the five key incentives facing the hospital [1]. As shown in Figure 3, the horizontal axis represents four different types of medical organization structures, the vertical axis represents five different reform dimensions [23,24]. The vertical axis of the Preker-Harding model represents five different reform dimensions of the medical alliance. These five dimensions are the key factors for measuring the behavior and performance of medical organizations, and are also an important reference for judging the organizational structure of medical organizations [24].

Decision right refers to the decision-making power of the medical alliance in its own operations and management process, such as financial management, infrastructure development, material procurement, capital operation, business management, etc [25]; Market competition refers to the degree to which the medical alliances compete with each other under the influence of market rules and obtain corresponding income by providing medical services required by patients [26]; Residual claim refers to the claim and control power of the management of the medical alliance [27]; Accountability refers to the responsibility and supervision of the medical alliance in providing medical services and accomplishing performance goals [28]; Social function refers to the social responsibility that the medical alliance should undertake [29], such as providing medical assistance to vulnerable groups and dispatching a health rescue team to the disaster area, especially the COVID-19.

From the above analysis, we can see that in the five dimensions of the vertical axis of the Preker-Harding model, the efficiency dimension includes three indicators: decision rights, market competition, residual claims. The fairness dimension includes two indicators: accountability, and social functions [10]. When the efficiency dimension and the fairness dimension are met simultaneously, the medical alliance can better realize social functions. Through the Preker-Harding model analysis, we found that the main problem of the China’s medical alliance is the matching of five dimensions and the improvement of the mechanism. Therefore, this requires further analysis.

In addition, this study selects the Preker-Harding model to analyze and evaluate the modes of the governance structure of the China’s medical alliance for the following reasons: 1) Based on the predicament of the China’s current medical alliance reform, this model can provide important theoretical guidance to help us clearly analyze the current development status of various medical alliance modes. And this model is a theoretical guidance model formed in order to help the reform of medical organizations in developing countries on the basis of analyzing successful cases in developed countries and drawing on institutional economics, regulatory management, and governance theory. Therefore, it has a good reference for the analysis of China’s medical alliances. 2) The model advocates the realization of the “legal person” administrative reform from the institutional reform structure. It summarizes the five key factors governing the reform of medical organizations: decision rights, market competition, residual claims, accountability, and social functions. These five factors are the key points for China’s current medical alliance reform difficulties. Therefore, using this model to analyze the mechanism of medical alliances plays a very important role in reform. 3) From 2011 to now, China’s call for “establishing the modern medical alliance” has become stronger and stronger. Its three most prominent features are institutionalized decentralization, effective governance, and scientific management. In addition, with the slowdown of China’s economic growth, China continues to encourage public institutions to carry out the “legal person” administrative reforms, which not only can promote the transformation of government functions, but also fully stimulate the innovation vitality of high-quality professionals through the establishment of new incentive mechanisms. At the same time, this can also promote the organization involved in reform to become a legal person with independent autonomy and equal rights and responsibilities, and become a real market entity. This is consistent with the core idea advocated by the Preker-Harding model.

## Methods

### Research Design

In order to study the governance mode of the Chinese medical alliance in depth, we conducted an interview-based case study guided by the framework of the Preker-Harding model and key incentives of the medical alliance. We strategically selected the four most representative medical alliance modes in China (The Shenzhen Luohu Medical Group, The Anhui Tianchang Medical Community, The Beijing Children’s Hospital Pediatric Union, The Telemedicine Collaboration Network Mode of the China-Japan Friendship Hospital). And summarized and evaluated the characteristics, core mechanism, actual impact, and experience of these modes. The selection was based on different sources: 1) National survey and analysis report on the implementation of the medical alliance policy of the National Health Commission of China, 2) The cities’ experience in implementing the National medical alliance policy and the summary report web pages, 3) Suggestions and references provided by relevant research experts. On this basis, in order to ensure the richness and diversity of this research, we have chosen as much as possible different modes in different regions to make the research and analysis process more thoughtful and enlightening. It should be noted that the reason why the above four modes are selected as the sampling plan is that these four modes represent the main development mode of China’s current medical alliance. This representativeness takes into account regional differences, medical level differences, and medical cognition differences. Table 1 lists some of the participants in this interview.

**Table 1:** Participants of This Interview.

The Shenzhen Luohu Medical Group	The Anhui Tianchang Medical Community	The Beijing Children’s Hospital Pediatric Union	The Telemedicine Collaboration Network Mode of the China-Japan Friendship Hospital

Leader of the government health management department	Leader of the government health management department	Leader of the government health management department	Leader of the government health management department
Public health coordinator	Public health coordinator	Public health coordinator	Public health coordinator
Chief executive officer	Chief executive officer	Council of Specialist Union	Director of telemedicine collaboration office
Hospital director of the alliance	Township hospital director	Hospital director of the alliance	Hospital director of the alliance
Alliance policy executive	Alliance policy executive	Alliance policy executive	Alliance policy executive
Alliance policy coordinator	Alliance policy coordinator	Alliance policy coordinator	Alliance policy coordinator
Alliance policy regulator and evaluator	Alliance policy regulator and evaluator	Alliance policy regulator and evaluator	Alliance policy regulator and evaluator
Doctor in the alliance	Doctor in the alliance	Doctor in the alliance	Doctor in the alliance
Community rehabilitation center doctor	Township hospital doctor		Head of technical department
Patient	Patient	Patient	Patient
Patient	Patient	Patient	Patient
Third-party news media	Third-party news media	Third-party news media	Third-party news media
Research expert	Research expert	Research expert	Research expert
Research expert	Research expert	Research expert	Research expert

### Data Collection

This research is an interview study of government health managers, CEOs and operating teams, research experts, patients, and third-party news media of the four selected medical alliance modes. Based on the experience of local medical alliance policy coordinators and executives, and combined with the advice provided by relevant research experts, we strategically selected informants. This interview was conducted and completed from November 2017 to March 2018, mainly in informants’ offices and hospitals under medical alliances. A total of 55 interviews were conducted with 55 informants (23 women and 32 men) representing different types of interviewees (Table 1). Each interview lasted between 30 minutes and 90 minutes. A total of 47 interviews were conducted in person, and 8 interviews were conducted by phone or video. This research used semi-structured interviews, which mainly covered the five themes of medical alliances: decision rights, market competition, residual claims, accountability, and social functions. This paper includes responses and analysis of all themes in the interview. All the interviews were recorded, and then voice and text transcription and analysis were performed. In addition, in order to supplement the interview data, we also conducted text analysis by reading and analyzing the annual summary reports, planning and regulatory evaluation materials of the four medical alliance modes, so as to promote the interview analysis process to have more material support.

### Data Analysis

Based on the data collection, the paper conducted an interview analysis. The thematic analysis was based on the analysis framework of the Preker-Harding model, and the four medical alliance modes were analyzed and compared. First of all, the interview material was read through, and the interview voice and text transcription materials were compiled. Secondly, the data set was coded in a systematic way to meet the theme dimension of the medical alliance (the coding of the data set mainly refers to editing the meaningful and characteristic data set information that meets the theme dimension of the medical alliance into ordered data units in a certain order). And the code was hierarchically summarized according to 3 levels (the 3 levels here were divided according to the analysis framework of the Preker-Harding model. The first-level subdivision codes are the macro framework of the Preker-Harding model, which mainly collects the overall views, opinions and attitudes of various interviewees on the medical alliance; The second-level subdivision codes are the theme dimension formed after adjustment, applicability, and improvement based on the Preker-Harding model. It mainly includes decision rights, market competition, residual claims, accountability, and social functions, and can systematically analyze the operation of the medical alliance mechanism and characteristics; The third-level subdivision codes are further improved and refined on the basis of the second-level subdivision codes. It mainly includes organizational changes, owner functions, investment decision-making rights, personnel management rights, financial management rights, social functions, government regulations, institutional environments. These eight indicators can not only comprehensively and directly analyze and evaluate different medical alliance modes, but because the indicators are more detailed, this study can collect more specific and detailed content from the interviewees. In particular, these 8 dimensions are developed in this article on the basis of the Preker-Harding Model, based on the principles of the Preker-Harding Model and combined with the characteristics of the China medical alliance to construct an evaluation index system.) (Table 2). It should be noted that, on the basis of voice and text transcription materials, the coding process was hierarchically coded based on the theme dimension of the medical alliance, and then codes were generated. In the process, this study discussed each coded transcription material and cross-checked the coded data to ensure that each code and content were properly classified, while coping with coding differences and other situations. Then, through further analysis, comparison, and integrated coding, it was refined and layered again according to the 3 levels mentioned above. Finally, the text content was analyzed in detail, and the interview results were systematically summarized and summarized according to the above analysis dimensions and levels. Wordstat software was used during the specific analysis process. In order to ensure the consistency of the text analysis results with the interview materials, this paper repeatedly reviewed and revised the interview themes and codes, and text analysis results.

**Table 2:** Data Coding Scheme and Overall Content Analysis Framework.

First-level Subdivision Codes	Second-level Subdivision Codes	Third-level Subdivision Codes

Macro framework of the Preker-Harding model (overall views, opinions and attitudes towards medical alliances)	Decision rights	Organizational changes Owner functions Investment decision-making rights Personnel management rights Financial management rights Social functions Government regulations Institutional environments
	Market competition	
	Residual claims	
	Accountability	
	Social functions	

### Quality Control and Ethical Considerations

Before the interview, the qualified interviewers were selected according to the purpose of qualitative interviews, and the relationship between the interviewers and the interviewees (relationship establishment, individual characteristics grasping) was ensured, and the research design was followed by the principle of “purposeful sampling”. At the same time, the informed consent form of the interview was issued in advance, following the ethical norms; The interview process required compliance with certain interview frames and conversational principles; At the data collation stage after the interview, the interviewers were retrained and required to transcribe in accordance with the collation requirements, and did not join the personal opinions.

## Results

### Basic Situation of the Four Medical Alliance Modes

Based on the development of various regions and actual conditions, China’s four most representative medical alliance modes have their own characteristics and operating mechanisms.

The Shenzhen Luohu Medical Group is an urban medical group alliance mode. Specifically, the group has integrated 6 public hospitals and 35 community health service centers in the jurisdiction, and has established 12 resource management centers (such as inspection center, imaging center, logistics distribution center, etc.). The chief of the group has a chief accountant and three vice presidents, and manages the group and its subordinate hospitals [30].

The Anhui Tianchang Medical Community is a county medical community alliance mode. Tianchang People’s Hospital, Tianchang Hospital of Traditional Chinese Medicine and Tiankang Hospital are the lead units respectively, and they have signed up with 36 primary medical institutions in the city to form three medical communities. This mode implements the responsibility system of the presidents of various hospitals under the leadership of the council [31].

The Beijing Children’s Hospital Pediatric Union is a cross-regional specialist alliance mode. The alliance was initiated by Beijing Children’s Hospital and consisted of 20 provincial and municipal children’s medical units. On this basis, the provincial and municipal children’s medical units, as regional children’s medical centers, form a medical alliance with the children’s medical units in the cities, counties and towns in the region. In terms of management structure, the union has two levels of operational mechanisms, one is the group council responsible for the strategic planning and overall development of the alliance, and the other is the expert committee responsible for medical technology research and services [32].

The Telemedicine Collaboration Network Mode of the China-Japan Friendship Hospital is a telemedicine collaboration network alliance mode, which does not involve the reform of the management system [33].

### Preker-Harding Model Analysis of Medical Alliance Mode

According to the five dimensions of the Preker-Harding model affecting organizational change, combined with interview content and text analysis, this study quantifies the five dimensions into the following eight evaluation indicators, so as to comprehensively evaluate and analyze the four typical medical alliance modes.

#### Organizational Changes

Organizational changes refer to the possibility, flexibility and degree of change in the management system and structure of the medical alliance. The Shenzhen Luohu Mode fulfills the government’s funder functions and regulatory responsibilities by establishing a legal entity independent of the health administrative department. This mode has the following four benefits: 1) It is conducive to clarifying the relationship between the medical group and the government, and realizing the “separation of management and operations” of public hospitals. 2) It helps to optimize the allocation of medical resources. By integrating medical resources such as hospitals and community medical services and rehabilitation centers in the jurisdiction, it can promote the integration of operation management, staffing and medical services. It can also promote the establishment of new compensation mechanisms. 3) This mode constitutes a community of interests by continuously improving the distribution mechanism of benefits. For example, the reform of the medical insurance payment method through “total budget management and balance retention” can improve the current situation of weak primary medical services. 4) It is conducive to establishing a medical service community. However, the Luohu Mode also has some problems in the reform process. First of all, under this corporate governance model of “separation of management and operations”, the functions between the government and the medical group are difficult to fully clarify. Secondly, the existing legal system lacks laws and regulations on the construction and supervision of medical alliances. At the same time, during the operation of this mode, the difference between medical endogenous variables and exogenous variables may lead to changes in the reimbursement rate of different levels of medical institutions within the group, the compensation mechanism for hospitalization deductible lines, the operating mechanism, and the main body of fees.

The Anhui Tianchang Mode is an operation and management mode. Through the establishment of the council, it can divide and integrate the medical service resources of counties and towns. This mode has the following two benefits: 1) This mode is conducive to the establishment of a division of labor and coordination mechanism, but also facilitates the formation of a community of interests and responsibilities among various medical service organizations. 2) This mode facilitates the integration of medical resources within the county, and truly promotes everyone to obtain reasonable basic medical services [34]. However, this mode also has some problems in the implementation process. First of all, the serious lack of medical and health service personnel hinders the development of the medical community. In addition, due to the limited level of medical technology and equipment in county-level hospitals, the ability to provide referrals for township health institutions and to solve specialized diseases and difficult diseases is lacking, and whether the effect of mentoring is obviously difficult to measure. Last but not least, the existing medical alliance construction mechanism and experience are still immature, and there are loopholes in the design of incentive system, interest distribution mechanism, and performance appraisal principles.

The Beijing Children’s Hospital Mode clarified the relationship between public hospitals, governments and investors through the introduction of third-party capital investment. This mode has the following three advantages: 1) In terms of management structure, the group council has created pressures for hospitals in the alliance by establishing a rigorous and dynamic assessment and elimination mechanism, thereby motivating them to achieve their goals as much as possible, which is conducive to improving the quality and level of medical services of various hospitals. 2) The alliance has formed a “sharing” mechanism (expert sharing, scientific research sharing, and clinical sharing), which are beneficial to all levels of hospitals to fill shortcomings and improve medical service capabilities. 3) The mode is able to integrate quality pediatric resources within the alliance. However, this mode also has obvious problems. First of all, many provincial and municipal public hospitals are difficult to break the control and constraints of the original administrative system. Whether the alliance can develop as expected still needs time. Secondly, there is a lack of a benign and long-lasting support mechanism, and the effectiveness of the assistance is also lacking in scientific evaluation.

The advantages of the China-Japan Friendship Hospital Mode are: it can effectively promote the realization of grading diagnosis and treatment, promote the level of primary medical services, and strengthen quality control through telemedicine information management. However, the China-Japan Friendship Hospital Mode also has some shortcomings in the reform. Firstly, a unified information platform is difficult to establish [35,36]. Since there is no uniform standard for telemedicine equipment in China, the interface functions, data formats and system compatibility of telemedicine in different regions are quite different. “Medical Information Islands” are not conducive to the realization of quality medical resources sharing [36]. Secondly, telemedicine lacks a unified evaluation system, and the system’s information security supervision is not enough [35]. Thirdly, telemedicine laws and regulations are not perfect.

#### Owner Functions

Owner functions are the funder functions, which are mainly embodied in the supervision of medical alliances and medical functions. The Luohu Medical Group is a corporate governance structure. The board of directors established on behalf of the government to exercise the decision-making power of medical groups, and the hospitals within the medical group have less residual claims. The group president is appointed by the board of directors and is responsible to the district government; The Anhui Tianchang Mode has established a government-led council, the personnel system is the responsibility of the presidents of the hospitals under the board of directors, and there is no right to appoint or dismiss the director; The Beijing Children’s Hospital Pediatric Union has set up the group council to be responsible for strategic planning and business development. This alliance has a relatively large residual claim by introducing third-party capital investment, but there is no hospital president’s right to appoint; The China-Japan Friendship Hospital Mode has a relatively large residual claim, but this mode doesn’t have the right to appoint hospital presidents.

#### Investment Decision-making Rights

Investment decision-making rights refer to the ability to make investment decisions for the development of medical alliances. The Luohu Mode council on behalf of the government to exercise investment decision-making power, the chief accountant under the group’s president to supervise and manage it; The council of Anhui Tianchang Mode mainly exercises hospital investment in large-scale equipment and infrastructure projects; The introduction of social capital in the Beijing Children’s Hospital Mode makes it have greater investment decision-making power; The China-Japan Friendship Hospital Mode has greater investment decision-making power, which is conducive to mobilizing the enthusiasm of hospitals and doctors.

#### Personnel Management Rights

Personnel management rights refer to the authority to appoint and manage personnel in the development of the medical alliance. In order to mobilize the enthusiasm of medical staff, the Luohu Mode is establishing a whole-staff appointment system; the Anhui Tianchang Mode mainly establishes a talent delivery system through the dynamic flow of county and township medical institutions; the Beijing Children’s Hospital Mode has less power in personnel management; the China-Japan Friendship Hospital Mode is a kind of cooperation and does not involve personnel management rights.

#### Financial Management Rights

Financial management rights refer to the power of financial management in the development of the medical alliance. The Luohu Medical Group is mainly responsible for the implementation of the budget, financial management rights are small; the Anhui Tianchang Mode takes the medical insurance fund as the link, mainly implementing the prepayment system, and the financial management rights are relatively small; the Beijing children’s hospital and the China-Japan Friendship Hospital Mode relies on social capital to establish a contractual relationship, and its autonomy in financial budgeting and auditing is relatively large.

#### Social Functions

Social functions mainly refer to the realization of the purpose of the medical alliance through various effective methods, the promotion of the realization of medical public welfare and the improvement of the level of primary medical services. The main social goal of the Luohu Medical Group is to sink high-quality medical resources and reduce the cost of patient diagnosis and treatment; This mode enhances the level of medical services and rehabilitation care in the grassroots community by establishing a community of health care services. And this goal is generally based on performance appraisal. The goal of the Anhui Tianchang Mode is to strengthen the training of grassroots medical institutions and promote the realization of graded diagnosis and treatment, this goal is achieved through accountability and assessment of the dean; the main social goal of the Beijing Children’s Hospital Mode is to improve the ability of technical research and treatment of intractable diseases; the goal of the China-Japan Friendship Hospital Mode is to promote the accessibility and fairness of primary health care services, mainly relying on third-party social capital participation and joint governance.

#### Government Regulations

Government regulations are to ensure that the services provided by the medical alliance are effective and reasonable. The Luohu Medical Group implements multi-party joint supervision. The group council acts as a legal representative to supervise the subordinate hospitals, the board of supervisors mainly supervises the operation of the alliance and the members of the board of directors, and the government health administrative department plays a regulatory role at the macro level; the Anhui Tianchang Mode is mainly supervised by the health administrative department; the Beijing Children’s Hospital Pediatric Union and the China-Japan Friendship Hospital Mode mainly implement responsibility through the health administrative department and industry supervision.

#### Institutional Environments

Institutional environments are an important policy factor to promote the construction of medical alliances. The four modes mentioned in this study have responded to the new round of medical reform calls by the Chinese government, and initiated the construction of medical alliances under the guidance of policies.

In summary, we obtained the results of the governance structure model of China medical alliance based on the Preker-Harding model and interview analysis, as shown in Table 3.

**Table 3:** Evaluation and Analysis of the Governance Structure Model of China Medical Alliance Based on Preker-Harding Model.

	The Shenzhen Luohu Medical Group	The Anhui Tianchang Medical Community	The Beijing Children’s Hospital Pediatric Union	The Telemedicine Collaboration Network Mode of the China-Japan Friendship Hospital

**Organizational changes**	Establish an independent legal entity, corporate governance structure model	Implement the president’s responsibility system under the leadership of the board of directors	Construct a contractual management model and establish a group council and expert committee	Implement an autonomous cooperative organization model, third-party social capital participation
**Owner functions**	The board of directors exercises the decision-making power	Financial management rights are small; no decree has the right to appoint	Introduce third-party capital investment, the residual claim is large; no decree	Introduce third-party social capital, have a large residual claim; no decree
**Investment decision-making rights**	The board of directors exercises investment decision-making power and is supervised by chief accountant	The council mainly exercises decisions on large equipment purchases and infrastructure projects in hospitals	Have greater investment decision-making power	Form a benign contractual relationship with third parties, greater investment decision-making power
**Personnel management rights**	Establish a full-time appointment system with greater personnel management rights	Dynamic flow of personnel and establishment of a talent delivery system	Small personnel management power	Small personnel management power
**Financial management rights**	Partial execution of budgetary rights	Partial financial management	Introduce social capital, financial management, revenue and expenditure have autonomy	Rely on social capital to establish a contractual relationship, greater autonomy
**Social functions**	Sink high-quality medical resources and reduce the cost of patient care	Promote two-way referral, grading diagnosis and treatment	Technical problems and the distribution of medical resources in difficult diseases	Establish a telemedicine service system
**Government regulations**	Joint supervision with the group council, the board of supervisors	Health administration to obtain supervision	Industry supervision, health sector supervision	Health administration and industry supervision
**Institutional environments**	Comply with the reform requirements of the medical insurance fund management	Respond to the call for a new medical reform	Comply with the national classification and treatment policy	Comply with the national classification and treatment policy

## Discussion

First of all, the essence of the relationship between the government as the owner and the medical alliance is the commission and adjustment of power and responsibility [37]. The four medical alliance modes in this study can reflect the government’s decentralization of some financial management rights, personnel management rights and residual claims in order to promote the rational allocation of quality medical resources in the dimensions of organizational change and owner functions. However, at present, the government’s supervision and evaluation of the medical alliance and the performance appraisal are not clear, and the accountability mechanism of the medical alliance in the medical public welfare has not been implemented. This has prompted the exploration and pilot of the reform of the medical alliance. For example, in the Shenzhen Luohu Mode, the government entrusted the owner’s rights and responsibilities to the Luohu Medical Group Legal Person. Because the government is difficult and costly to perform the functions of the owners of multiple medical institutions at the same time, entrusting the authority and responsibility to the Luohu Medical Group Legal Person can greatly reduce the burden on the government, and is also conducive to the medical alliance to achieve social functions and improve internal performance.

The government as a regulator has a normative and universal regulation of the medical alliance. The normative nature of government regulation is regulated in accordance with laws and regulations. The universality of government regulation is that the supervision of various medical alliances should reflect fairness and impartiality. However, at present, China’s laws and regulations on medical supervision are not perfect, so the government’s supervision of the medical alliance is not in place.

The reform of the medical alliance requires the government to clarify the functional positioning of the medical alliance and determine a reasonable compensation system. Medical alliances need to consider whether the social function of medical alliances is impaired when conducting corporate or contractual governance changes [38]. Of course, in order to promote the realization of grading diagnosis and treatment and the sharing of quality medical resources, the government needs to give medical alliances a certain degree of financial management autonomy and reasonable compensation. However, at present, the government’s responsibility for financial compensation is lacking, which may cause medical alliances to lose balance between public welfare and efficiency.

**Figure 4:**
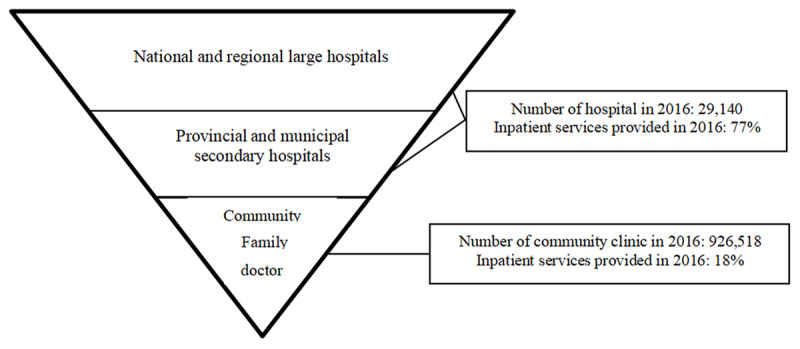
The “Inverted Triangle” Model of China’s Medical Security Supply.

**Figure 5:**
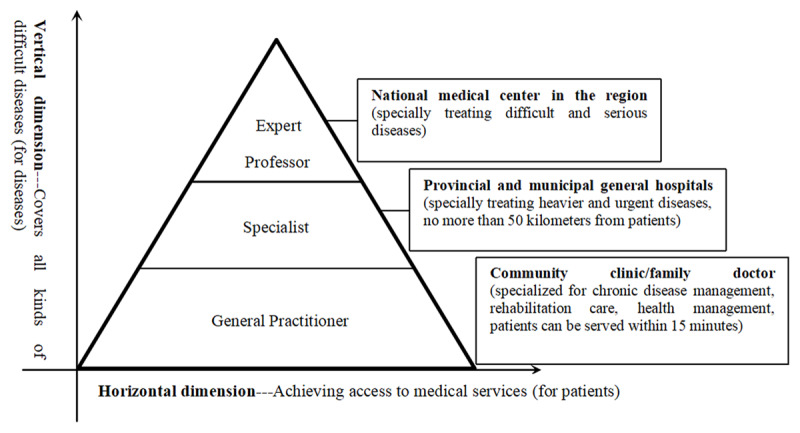
The “Positive Triangle” Model of China’s Medical Security Service.

Based on the research results, we found that the four medical alliance modes in China have differences in many dimensions, such as the ability of organizational changes, the size of the owner functions, and the realization of social functions. These differences between the medical alliance modes directly affect its ability to optimize the allocation of medical resources. As mentioned earlier, although China’s medical alliances have many forms, most medical alliances have the same purpose, that is, to promote hierarchical diagnosis and treatment, optimize the allocation of medical resources, improve the service level of primary medical institutions, and comprehensively improve the health management level and chronic disease management level. For example, when COVID-19 broke out, China’s medical alliance played a huge role. In many regions, community medical alliances provided free COVID-19 education, protection guidance, temperature and body testing to local residents. Confirmed or suspected patients shall be quarantined and reported to the higher-level health department. In the process of epidemic prevention, some community medical institutions have maintained good cooperation with other medical institutions in medical alliances (network contact, online remote command, deployment of medical personnel and materials, etc.) to facilitate timely transmission of epidemic prevention and control information and the reasonable regulation of medical resources and so on. However, China’s medical resources are still very scarce. Due to China’s large population and increasingly serious aging, coupled with the imbalance in the allocation of medical resources between regions, China’s medical security undertaking is facing huge difficulties. Under such circumstances, the development of China’s medical alliance needs to rely on the “positive triangle” model of medical security services. At present, China’s medical resources are allocated in an “inverted triangle” mode, most patients are concentrated in medical centers and provincial medical institutions, most medical resources and patients are concentrated in medical centers and provincial medical institutions, the siphon effect is abnormally obvious. However, most diseases can be cured in grassroots clinics or community health management center. There is a huge contradiction between this “inverted triangle” medical resource supply model (Figure 4) and the “positive triangle” medical resource demand. Therefore, it is of great significance to construct a “positive triangle” medical service governance model (Figure 5), especially for the future development of the primary medical alliance (sinking part of the high-quality medical resources into the primary medical alliance can not only meet the general medical needs of most people, but also promote the development of primary medical institutions and the reasonable allocation of medical resources). Of course, this requires the intervention of medical insurance payment methods, such as DRGs-PPS (Diagnosis Related Groups-Prospective Payment System), Pay-For-Value, etc. By establishing quantitative indicators (based on GDP or other economic development indicators to set the growth rate of medical insurance funds, establish an annual budget fund formation mechanism, use the disease group points method to calculate the cost coefficient, etc.), and improving the intelligent supervision system, it is possible to promote the rational use of medical insurance funds, and at the same time promote the rationalization of the medical resource supply mode, so as to truly achieve graded diagnosis and treatment and improve the health of residents.

There are some limitations in the research. On the one hand, only the four typical medical alliance modes in China are selected. This does not summarize the pattern of medical resource allocation in China. However, the essential purpose and trend of all alliances are the same, which we have explained in the first part of the article. On the other hand, due to the limitations of the research perspective and the time constraints of the research process, the specific factors and background information of some medical alliance modes are not rich enough, and the future needs to be further deepened and enriched.

## Conclusions

This study concludes that different medical alliance modes have their own advantages and disadvantages, but the corporatization mode has more obvious advantages. In addition, in order to promote the realization of grading diagnosis and treatment and the effective sharing of quality medical resources, China should establish the “Positive Triangle” model of medical alliance’s governance.
